# Risk Factors for Snoring in Two Canadian First Nations Communities

**DOI:** 10.3390/clockssleep1010011

**Published:** 2019-01-18

**Authors:** James A. Dosman, Chandima P. Karunanayake, Kathleen McMullin, Sylvia Abonyi, Donna Rennie, Joshua Lawson, Shelley Kirychuk, Niels Koehncke, Jeremy Seeseequasis, Laurie Jimmy, Vivian R. Ramsden, Mark Fenton, Gregory P. Marchildon, Malcolm King, Punam Pahwa, for the First Nations Lung Health Project Team

**Affiliations:** 1 Canadian Centre for Health and Safety in Agriculture, University of Saskatchewan, 104 Clinic Place, Saskatoon, SK S7N 2Z4, Canada; 2 Department of Medicine, College of Medicine, University of Saskatchewan, 103 Hospital Drive, Saskatoon, SK S7N 0W8, Canada; 3 Department of Community Health and Epidemiology, College of Medicine, University of Saskatchewan, 107 Wiggins Road, Saskatoon, SK S7N 5E5, Canada; 4 College of Nursing, University of Saskatchewan, 104 Clinic Place, Saskatoon, SK S7N 2Z4, Canada; 5 Community A, PO Box 96, Duck Lake, SK S0K1J0, Canada; 6 Department of Academic Family Medicine, University of Saskatchewan, West Winds Primary Health Centre, 3311 Fairlight Drive, Saskatoon, SK S7M 3Y5, Canada; 7 Division of Respirology, Critical Care and Sleep Medicine, Department of Medicine, College of Medicine, University of Saskatchewan, 103 Hospital Drive, Saskatoon, SK S7N 0W8, Canada; 8 Institute of Health Policy, Management and Evaluation, University of Toronto, Suite 425, 155 College Street, Toronto, ON M5T 3M6, Canada

**Keywords:** First Nations people, snoring, overweight, obesity, smoking, dampness

## Abstract

Snoring may be an important predictor of sleep-disordered breathing. Factors related to snoring among First Nations people are not well understood in a population with high rates of smoking and excess body weight. An interviewer-administered survey was conducted among 874 individual participants from 406 households in 2012 and 2013 in two Canadian First Nations communities. The survey collected information on demographic variables, individual and contextual determinants of respiratory health and snoring (classified as present versus absent) and self-reported height and weight. Multiple logistic regression analyses were conducted to examine relationships between snoring and potential risk factors adjusting for age and sex. Snoring was present in 46.2% men and 47.0% women. Considering body mass index, 259 people (30.3%) were overweight and 311 (36.4%) were considered obese. The combined current/former smoking rate was 90.2%. Being overweight, obesity, sinus trouble, current smoking status and former smoking were significantly associated with snoring. Exposure to home dampness and mold were suggestive of an association with snoring. To the degree that snoring may be a predictor of possible sleep-disordered breathing, these results indicate that environmental conditions such as smoking and home exposures may be important factors in the pathogenesis of these conditions.

## 1. Introduction

Snoring is a common symptom of potential sleep disordered breathing (SDB) with risk factors including age, body weight and smoking [1,2,3,4]. SDB has been associated with a number of outcomes including sleepiness [5,6], obstructive sleep apnea (OSA) [7,8], heart disease [8,9] possibly mediated by OSA [10], and injury [11]. A number of studies have indicated that there may be factors specific to sleep in North American Indian populations [12,13,14]. O’Connor et al. showed there is an increased risk of snoring in American Indian women and an increased risk of breathing pauses during sleep in both men and women versus non-Hispanic White populations [12]. Redline et al. reported lighter sleep in American Indians [13]. Froese et al. reported that snoring in three Indigenous North American groups in British Columbia was strongly related to sleepiness [14]. Mihaere et al. reported an unadjusted increased risk ratio for snoring among New Zealand Māori versus non-Māori populations and a higher prevalence of obstructive sleep apnea that appeared attributable to body habitus [15]. In this report, we explore factors relating to snoring as a possible indicator of SDB in two First Nations communities in Saskatchewan. They comprise a population characterized by youthfulness, high body weight, high smoking rates and exposure to indoor air quality issues. We used the population health framework [16] to assess individual and contextual factors relating to the outcome of snoring in these communities.

## 2. Results

Snoring was prevalent in 47.0% of women, 46.2% of men and 46.6% overall in our study. Demographic data (Table 1) show that the population is young (mean age ± SD = 35.2 ± 14.4 years) and is characterized by high rates of overweight (30.3%) and obesity (36.4%). The populations are characterized by high rates of current smoking (77.7%) and former smoking (12.5%).

Bivariable analyses, as shown in Table 2, demonstrated a number of putative associations with snoring; these include age (OR 2.13, 95% CI (1.37, 3.29)), body mass index (BMI) (obese: 3.18 (2.26, 4.48); overweight: 1.77 (1.27, 2.46)), smoking status (current smoker: 1.84 (1.14, 2.95); former smoker: 2.23 (1.27, 3.94)), sinus trouble (2.06 (1.52, 2.79)), heart problems (1.66 (1.05, 2.64)), shortness of breath (1.36 (1.04, 1.76)), cough (1.45 (1.11, 1.91)), wheeze (1.80 (1.28, 2.53)), house dampness and mold (1.52 (1.13, 2.04)), and house in need of repair (major repair: 1.53 (1.11, 2.11); minor repair: 1.37 (0.95, 1.98)).

As shown in Table 2, multivariable logistic regression analysis taking into account household clustering, shows persistent associations for age (1.80 (1.13, 2.85)), sex (male: 1.35 (1.00, 1.83)), BMI (obese: 3.16 (2.18, 4.60); overweight: 1.75 (1.24, 2.46)), smoking status (current smoker: 2.36 (1.46, 3.82); former smoker: 2.32 (1.30, 4.14)) and sinus trouble (1.86 (1.35, 2.57)). Environmental conditions as represented by home dampness and mold (1.33 (0.97, 1.84); *p* = 0.078) are suggestive of a relationship with snoring. Interactions between potential effect modifiers were examined and were not significant.

## 3. Discussion

The results of this study demonstrate that age, body weight and smoking are related to snoring among First Nations people, findings that have previously been shown in primarily Caucasian populations [1,2,3,4]. The striking feature of these results among First Nations people is that these findings occur among populations that are characterized by youthfulness, having high body weight, almost universal present or past smoking, many of whom live in houses characterized by having water and mold damage.

It is not clear from our data if being a First Nations person is a risk factor for snoring. Young et al., in the Sleep Heart Health Study, reported that the frequency of snoring in participants with an apnea/hypopnea index (AHI) ≥ 15 was higher among American Indians (23%) than among White people (17%) [17]. In the multiple logistic regression analyses, when snoring loudness was added to a model consisting of age, sex, race and body habitus, the odds ratio for American Indians versus White participants was 1.26 (1.00–1.60) [17]. Young et al. also determined that the increased risk for SDB (AHI ≥ 15) for American Indians versus White participants (1.70 (1.37–2.11)) could be explained as result of increased body habitus measurements (height, weight, hip circumference) [17]. Froese et al. reported on sleep patterns in a population of Indigenous participants in British Columbia, Canada [14], the mean age of which was older (43.2 years) than was that of the populations we studied (35.2 years). They listed the prevalence of “frequent snoring” as 36.0% (versus “do you snore” 46.6% in our study). Indirect evidence of enhanced snoring risk among New Zealand’s Māori Indigenous people is suggested by Gander et al. who related it to an increased risk of high Epworth Sleepiness Scale score [18]. Recognizing that Indigenous populations are not homogenous, it is possible that the American Indians included in the studies by Young et al. [17] might have more common lineages with the Cree Nations that we studied, as compared to the Pacific Māori populations [18].

The populations from the communities that we studied (mean age 35.2 years) are much younger than non-Indigenous populations in rural Saskatchewan described by Gjevre et al. (55.0 years) [19] and Pahwa et al. (52.0 years) [20]. The prevalence of snoring in the two communities was related to age and BMI. However, age and BMI were not strongly correlated (*R* value 0.269), indicating that both age and BMI may be independently related to snoring. Multivariable analysis demonstrates this independent influence of age and body weight on the outcome of snoring among these First Nations people. In addition, we examined for interactions and these were not significant.

Recognizing that snoring and OSA are not the same, evidence from non-Indigenous populations indicate that there is a two to three times increase in risk for OSA in men compared to women [4]. We did not see the same trend in the symptom of snoring, as multivariable analysis showed only a modest increase in snoring in men as compared to women in our study (OR 1.35 (1.00–1.83)) (*p* < 0.049). This may be the case because the women had higher mean value for BMI than did men (30.1 versus 26.8) with more women (46.6%) than men (26.1%) fitting the criteria for obesity. Smoking is also associated with snoring [1,2], and with 90.2% of the populations in our study being current (77.7%) or former (12.5%) smokers, multivariable analysis demonstrated strong associations for both being a current smoker (OR 2.36) and an ex-smoker (OR 2.32).

We were interested in the possible role of environmental exposures on the symptom of snoring. Smoking may be considered an environmental exposure and is a strong independent risk factor for snoring among the people who participated in our project. We were similarly interested in the effect of indoor household exposures. Poor housing conditions [21] among First Nations communities are associated with an increased risk of asthma, allergies, severe respiratory infections and tuberculosis [22,23,24,25,26].

Of the data that we elicited, both the variables of home dampness and mold, as well as house in need of repairs, showed associations with snoring in the bivariable analysis. In the multivariable analysis, house dampness and mold continued to show a trend with snoring. This association is not well established and it could be a possible association between snoring and allergy to mold. In the Seven US Urban Areas Study, atmospheric air pollution was associated with evidence for OSA, possibly because of inflammation in the oropharynx [27]. This could be the case for snoring among participants in our study and thus snoring and sleeping environment warrants further investigation.

We ascertained that doctor diagnosed conditions and symptoms related to the heart and lungs were associated with snoring in the bivariable analysis. In the final model, sinus trouble remained strongly associated with snoring, which has previously been described [28]. We postulate that sinus trouble associated with nasal congestion could be related to environmental exposures in the houses, and in turn cause or aggravate snoring in this population. In contrast to previously reported studies, we did not observe a relationship between alcohol consumption and snoring [29,30].

This study has a number of strengths including the size of the groups participating, the strengths of associations of age, sex, BMI and smoking with snoring, and the possibility that environmental exposures may be risk factors for SDB in this unique population. There are ways in which these findings might be enhanced and there are few significant limitations in this study. Firstly, as these are cross-sectional observations, causation can only be speculative. Another potential limitation is that this study is based on self-reported data. Since we selected these two First Nations Communities based on accessibility, potential selection biases are a possibility. Moreover, the study participants are not representative of the general population of other North American First Nations. Therefore, these results cannot be generalized to other North American First Nations. The original purpose of the study was to examine the predictors of respiratory health in First Nations populations and the investigation of sleep was a secondary consideration. Another major limitation was that major sleep variables and screening scores for sleep apnea were not collected in this study. In addition, despite best efforts, there may be cultural barriers to participants fully understanding some of the questions, which may influence our findings.

Given the diversity of First Nations communities, the findings of this paper may not be generalized to other communities or populations. However, this should not be considered a limitation as the findings may be of interest in populations and contexts that do share some of the characteristics described here. In addition, the methodology is transferable and adaptable to similar contexts. Future comparable research elsewhere could enrich our understanding of the relationship between snoring and risk factors among other First Nations communities across Saskatchewan and Canada.

## 4. Materials and Methods

### 4.1. Study Design

The First Nations Lung Health Project (FNLHP) is a prospective cohort study design being conducted using interviewer-administered surveys in two phases; baseline and follow-up [31]. The baseline survey was completed between 2012 and 2013. Analyses in this report are based on the cross-sectional survey data. The detailed methodology has been described [17]. In brief, data were collected by surveys from First Nations adults living in two Cree First Nations communities in north-central Saskatchewan. The communities were selected based on previously established relationships. A Decision Makers Council consisting of band councilors, elders and youth assisted in guiding the surveys and research. The Biomedical Research Ethics Board of the University of Saskatchewan approved the study (Certificate No. Bio #12-89). The work adheres to guidelines of the Government of Canada, Tri-Council Policy Statement 2—Chapter 9—Research Involving the First Nations, Inuit and Métis Peoples of Canada [32]. Written consent was obtained from all participants.

### 4.2. Variables

The questionnaire collected information on individual and contextual factors and self-reported snoring (outcome variable) as descried below.

Primary Health Outcome: The outcome of interest was self-reported snoring based on the question “Do you snore (yes/no/do not know)?”

Individual Factors and Covariates: The demographic variables; age, sex, body mass index, cigarette smoking and alcohol consumption were collected from technician assisted questionnaire surveys. Medical conditions such as ever being doctor-diagnosed with chronic bronchitis, asthma, heart problems and sinus trouble were ascertained. Respiratory symptoms of cough, phlegm, wheezing and shortness of breath were assessed.

Contextual Factors: The principal contextual factors considered with the outcome of snoring were socioeconomic status (money left at the end of the month) and environmental conditions (presence of proper ventilation such as the use of an air conditioner, humidifier or dehumidifier, pets inside home and indoor smoking). The environmental variable of house dampness or mold was derived from positive responses to any of the following questions: “During the past 12 months, has there been water or dampness in your house from broken pipes, leaks, septic tank, heavy rain, or floods?”; “Does your house have any damage caused by dampness?”; “Does your house frequently have a mildew/moldy odor or musty smell?”; and “Are there signs of mold or mildew in any living areas in your house?”. Houses in need of repairs was assessed with three options: Major repairs needed; minor repairs needed; only regular maintenance is required.

### 4.3. Statistical Analyses

Statistical analyses were conducted using SPSS version 24 (IBM SPSS Statistics for Windows. Armonk, NY: IBM Corp., 2015). Frequencies were computed for all variables. Chi-square tests were used to determine the bivariable association of snoring prevalence with the independent variables of interest. Logistic regression models were used to predict the relationship between a binary presence of snoring (yes or no) and a set of explanatory variables. A multilevel logistic regression modeling approach using generalized estimating equations with individuals (1st level) nested within households (2nd level), was utilized to evaluate the effects of both contextual and individual factors after adjustment for covariates of interest. This accounts for the within household dependencies that occur in the analysis due to multiple people from the same household. A series of multi-level models were fitted to determine whether potential risk factors, confounders, and interactive effects (e.g., individual and contextual risk factors) contributed significantly to explanatory variables. Based on bi-variable analysis, variables with *p* < 0.20 were candidates for the multivariate model. All variables that were statistically significant (*p* < 0.05), as well as important contextual factors (environmental conditions), were retained in the final multivariable model. Interactions between potential effect modifiers were examined and were retained in the final model if the *p*-value was <0.05. The strengths of associations were presented by odds ratios (OR) and their 95% confidence intervals (CI) [33,34]

## 5. Conclusions

We have identified convincing relationships between age, sex, BMI, smoking status and possibly environmental exposures on the one hand, and snoring as a possible marker for SDB on the other hand in these First Nations communities. These finding point to the need for primary care providers to be aware of the possible relationships involved in First Nations patients presenting with snoring, especially early in life. There is an urgent need for a more in-depth evaluation of SDB and its principle determinants including home exposures in Canadian First Nations communities. Such studies should be considered in the context of demonstrated systemic inequities with regard to access to care for obstructive sleep apnea among Indigenous people [35].

## Figures and Tables

**Table 1:** Demographic characteristics and snoring prevalence of the study populations *.

Variable	Men *N* = 431 (49.3%)	Women *N* = 443 (50.7%)	All *N* = 874
Age, in years: mean ± SD	34.4 ± 14.3	36.1 ± 14.4	35.2 ± 14.4
Body mass index: mean ± SD	26.8 ± 6.1	30.1 ± 6.9	28.4 ± 6.7
Obese	111 (26.1)	200 (46.6)	311 (36.4)
Overweight	138 (32.4)	121 (28.2)	259 (30.3)
Normal	177 (41.5)	108 (25.2)	285 (33.3)
Smoking Status			
Current smoker	340 (78.9)	338 (76.5)	678 (77.7)
Ex-smoker	49 (11.4)	60 (13.6)	109 (12.5)
Never smoker	42 (9.7)	44 (10.0)	86 (9.9)
Snoring			
Yes	199 (46.2)	208 (47.0)	407 (46.6)
No	232 (53.8)	235 (53.0)	467 (53.4)

* Column percentages were reported where applicable.

**Table 2:** Regression analysis of the relationship between various factors and snoring outcome.

Variable	Snoring		OR_unadjusted_^†^ (95% CI)	OR_adjusted_^†^ (95% CI)
	Yes n (%)	No n (%)		
**Demographics**				
Age Group, in years				
>55	57 (14.0)	33 (7.1)	2.13 (1.37, 3.29) **	1.80 (1.13, 2.85) *
≤55	350 (86.0)	434 (92.9)	1.00	1.00
Sex				
Male	199 (48.9)	232 (49.7)	0.98 (0.75, 1.27)	1.35 (1.00, 1.83) *
Female	208 (51.1)	235 (50.3)	1.00	1.00
Body Mass Index				
Obese	188 (47.0)	123 (27.0)	3.18 (2.26, 4.48) **	3.06 (2.18, 4.60) **
Overweight	119 (29.8)	140 (30.8)	1.77 (1.27, 2.46) **	1.75 (1.24, 2.46) **
Not overweight or obese	93 (23.2)	192 (42.2)	1.00	1.00
Smoking Status				
Current smoker	322 (79.1)	356 (76.4)	1.84 (1.14, 2.95) **	2.36 (1.46, 3.82) **
Ex-smoker	57 (14.0)	52 (11.2)	2.23 (1.27, 3.94) **	2.32 (1.30, 4.14) **
Never smoker	28 (6.9)	58 (12.4)	1.00	1.00
Money left at the end of the month				
Not enough	196 (50.1)	210 (48.1)	1.03 (0.74, 1.44)	-
Just enough	87 (22.3)	110 (25.2)	0.87 (0.58, 1.29)	-
some	108 (27.6)	117 (26.8)	1.00	-
Alcohol consumption				
Regular	94 (23.2)	122 (26.3)	0.85 (0.63, 1.14)	-
Never or not regular	312 (76.8)	342 (73.7)	1.00	-
**Medical conditions**				
Ever Chronic Bronchitis				
Yes	33 (8.1)	24 (5.1)	1.61 (0.98, 2.66)	-
No	374 (91.9)	443 (94.9)	1.00	-
Ever Asthma				
Yes	71 (17.4)	78 (16.7)	1.05 (0.73, 1.50)	-
No	336 (82.6)	389 (83.3)	1.00	-
Ever Sinus trouble				
Yes	143 (35.1)	97 (20.8)	2.06 (1.52, 2.79) **	1.86 (1.35, 2.57) **
No	264 (64.9)	370 (79.2)	1.00	1.00
Ever Heart problems				
Yes	50 (12.3)	36 (7.7)	1.66 (1.05, 2.64) *	-
No	357 (87.7)	431 (92.3)	1.00	-
Shortness of breath				
Yes	231 (56.8)	230 (49.3)	1.36 (1.04, 1.76) *	-
No	176 (43.2)	237 (50.7)	1.00	-
Cough				
Yes	194 (47.7)	180 (38.5)	1.45 (1.11, 1.91) **	-
No	213 (52.3)	287 (61.5)	1.00	-
Phlegm				
Yes	206 (50.6)	222 (47.5)	1.14 (0.88, 1.48)	-
No	201 (49.4)	245 (52.5)	1.00	-
Wheeze				
Yes	329 (80.8)	328 (70.2)	1.80 (1.28, 2.53) **	-
No	78 (19.2)	139 (29.8)	1.00	-
Any injury past 12 months				
Yes	89 (21.9)	110 (23.6)	0.92 (0.67, 1.26)	-
No	318 (78.1)	357 (76.4)	1.00	-
**Environmental conditions**				
Smoke inside home				
Yes	219 (53.8)	247 (52.9)	1.04 (0.79, 1.37)	-
No	188 (46.2)	220 (47.1)	1.00	-
Any pet in home				
Yes	136 (33.4)	147 (31.5)	1.09 (0.82, 1.45)	-
No	271 (45.9)	320 (54.1)	1.00	-
Dehumidifier in home				
Yes	32 (7.9)	55 (11.8)	0.65 (0.40, 1.04)	-
No	375 (92.1)	412 (88.2)	1.00	-
Humidifier in home				
Yes	58 (14.3)	66 (14.1)	1.01 (0.71, 1.43)	-
No	349 (85.7)	401 (85.9)	1.00	-
Air conditioner in home				
Yes	107 (26.3)	110 (23.6)	1.15 (0.85, 1.56)	-
No	300 (73.7)	357 (76.4)	1.00	-
House dampness and mold				
Yes	302 (74.2)	306 (65.5)	1.52 (1.13, 2.04) **	1.33 (0.97, 1.84) ^#^
No	105 (25.8)	161 (34.5)	1.00	1.00
House in need of repair				
Yes, major repairs	175 (43.0)	170 (36.4)	1.53 (1.11, 2.11) *	-
Yes, minor repairs	112 (27.5)	120 (25.7)	1.37 (0.95, 1.98)	-
No, only regular maintenance required	120 (29.5)	177 (37.9)	1.00	-

† Bivariable and multivariable regression models were fitted using Generalized Estimating Equations taking into account the household clustering. ^#^ *p* < 0.10; * *p* < 0.05; ** *p* < 0.01.

## Abbreviations

SDB	Sleep Disordered Breathing
OSA	Obstructive Sleep Apnea
BMI	Body Mass Index
AHI	Apnea/Hypopnea Index
FNLHP	First Nations Lung Health Project
OR	Odds Ratio
CI	Confidence Interval
